# Relationship between Acceleration in a Sit-To-Stand Movement and Physical Function in Older Adults

**DOI:** 10.3390/geriatrics8060123

**Published:** 2023-12-16

**Authors:** Korin Tateoka, Taishi Tsuji, Takuro Shoji, Satoshi Tokunaga, Tomohiro Okura

**Affiliations:** 1 Doctoral Program in Physical Education, Health and Sport Sciences, University of Tsukuba, Tsukuba 305-8571, Japan; 2 Institute of Health and Sport Sciences, University of Tsukuba, Tokyo 112-0012, Japan; tsuji.taishi.gn@u.tsukuba.ac.jp (T.T.); okura.tomohiro.gp@u.tsukuba.ac.jp (T.O.); 3 Doctoral Program in Public Health, Degree Programs in Comprehensive Human Sciences, Graduate School of Comprehensive Human Sciences, University of Tsukuba, Tsukuba 305-8571, Japan; takuro11131113@gmail.com (T.S.); s2130445@u.tsukuba.ac.jp (S.T.)

**Keywords:** acceleration parameters, chair-rise, performance test

## Abstract

Acceleration parameters in sit-to-stand (STS) movements are useful for measuring lower-limb function in older adults. The purpose of this study was to examine the relationship between acceleration in STS movements and physical function and the test-retest reliability of acceleration parameters in older adults. We performed cross-sectional analyses on 244 older adults including 107 men (mean age: 77.4 ± 4.7) and 137 women (mean age: 75.6 ± 5.3). Four acceleration parameters were measured in STS movements: maximum acceleration (MA), maximum velocity (MV), maximum power (MP), and stand-up time (ST). Good intraclass correlation coefficients (ICC > 0.70) were observed for all parameters. For the acceleration parameters, MA, MV, and MP were relatively strongly associated with the 5-time STS test (men: r = −0.36~−0.47; women: r = −0.37~−0.45) and the timed up and go test (men: r = −0.39~0.47, women: r = −0.43~−0.51): MP was also strongly associated with grip strength (men: r = 0.48, women: r = 0.43). All acceleration parameters were poorer in participants reporting mobility limitations than in those reporting no mobility limitations. These findings support the usefulness of sensor-based STS measurement. The system is expected to be useful in various settings where care prevention is addressed.

## 1. Introduction

Decreased lower extremity muscle strength in older adults is known to be an independent factor in the occurrence of serious events in old age, such as falls [1], decreased independence [2], and high mortality rates [3]. As a result, many attempts have been made to evaluate lower limb muscle strength in older adults, and various methods have been adopted [4,5,6].

Recent attempts have evaluated lower limb muscle strength and lower limb muscle power using acceleration in sit-to-stand (STS) movement [7,8,9]. In this method, a participant was asked to stand up while wearing a small sensor such as an accelerometer, and the evaluation was based on the parameters obtained from the sensor. Specifically, acceleration parameters (maximum acceleration and speed) and time parameters (time to complete the action) were used. The advantages of this measurement method include its ability to evaluate the exertion of muscle strength during daily activities [10], and the fact that the sensor itself is small and portable [11].

The relationship between acceleration parameters and a lower limb muscle strength evaluation index (knee extension muscle strength) in STS movement has already been examined [12] and validated and is being studied as a lower limb muscle strength evaluation method. In addition, studies on acceleration parameters and factors that influence the quality of life of older adults, such as physical function [13] and experience of falling [14,15], have also been reported. Regterschot et al. [16]. examined the relationship between acceleration parameters and ground reaction force during STS movement and found that acceleration assessment during STS movement could be an alternative method for assessing physical function in clinical settings. However, in various sites and fields working to prevent the need for nursing care, various performance tests are used to assess all types of physical function, in addition to lower limb muscle strength and muscle power assessment.

In addition to performance tests, older adults’ physical function can also be assessed through questionnaires and interviews. It is recommended to consider performance tests and questionnaires as distinct but complementary methods [17]. In addition, they have been reported to correlate with each other [18]. Furthermore, according to the model proposed by Glass, functions are divided into three categories, and he advocates that a distinction must be made between performance and function, since this creates a discrepancy between the two [19]. In other words, in order to clarify the usefulness of the acceleration assessment in STS movements, it is necessary to examine the relationship between the physical function assessed by the performance test and the physical function assessed by the questionnaire, respectively. Regterschot et al. also examined the association between acceleration parameters and physical function (timed up and go, 5-time STS time, and stair climbing test) in 36 older adults and found significant associations. Van Lummel et al. examined the association between subjective physical function and STS movement in older adult participants and reported that sensor-based recorded times were more strongly associated than manually recorded times [20].

However, due to the nature of studies that deal with physical fitness data such as acceleration, laboratory-based verification is the main focus, and the limitations are that the number of subjects is small and the studies focus on specific subjects such as facility residents and female older adults. Since previous studies have examined the relationship between ground reaction force during chair rise and physical function by gender and reported gender differences in the strength of the relationship [21], it is necessary to conduct a similar study on acceleration in STS movements aimed at evaluating lower limb muscle strength in the older adults. In addition, while it is important to prevent the progression of serious illness among the older adults staying in hospitals and facilities, it is also important to detect and appropriately deal with those with functional decline at an early stage [22]. Therefore, it is necessary to examine the usefulness of acceleration in STS movements in healthy older adults. Furthermore, in previous studies, performance items were limited to lower extremity function [13]. In addition to lower limb function, upper limb muscle function and other functions are necessary for the older adults to smoothly carry out their daily lives, and therefore, it is necessary to include these in the study [23,24].

Although test-retest reliability in acceleration parameters in STS movements has been examined in the past, the subjects were limited to males [25]. In addition, compared to previous studies [8] that reported good reliability, the reliability of the acceleration parameter used in this study needs to be confirmed once again, since this study used a measurement device with a high sampling period.

This study aimed to clarify the relationship between acceleration parameters and physical function based on performance tests (objective assessment) and questionnaires (subjective assessment) in STS movements in older adults randomly selected from the Basic Resident Registers to increase the representativeness and sample size of the older adults living in the community. In addition, the test-retest reliability was examined. This study hypothesized that each of the acceleration parameters in STS movement would show good test-retest reliability, and that better values for each parameter would be associated with better physical function. It has also been reported that kinematic (acceleration, velocity) and kinetic (power) parameters are more strongly associated with lower limb muscle strength, lower limb muscle power and falls than time parameters (rise time) in acceleration parameters in STS movement [15,26]. We hypothesized that kinematic and kinetic parameters would be more strongly associated with physical function than temporal parameters in this study.

## 2. Materials and Methods

### 2.1. Participants

This study included community-dwelling older individuals who participated in the 2020 Kasama Longevity Health Examination. This open cohort study was initiated in Kasama City in 2009 [27]. Participants were randomly selected from the Basic Resident Registration Network System according to the following criteria: (i) age 65 years or older, (ii) living in Kasama City, and (iii) not receiving long-term care insurance (independent in activities of daily living). In 2020, 840 people were informed by mail, and 252 (30.0%) participated in the survey. Although there were no exclusion criteria for this study, of the 252 individuals, 3 with missing questions and 5 who required assistance in getting up from a chair were excluded, leaving 244 for the final analysis. Acceleration measurements were conducted again on 12 participants, and the results were analyzed to examine reliability. This study was approved by the Ethics Committee of the University of Tsukuba (ref. no. Tai 30-5). All participants provided written informed consent to participate in the study, which was conducted in accordance with the Declaration of Helsinki.

### 2.2. Measurement Items

#### 2.2.1. Acceleration Parameters in STS

##### Procedures

**Figure 1:**
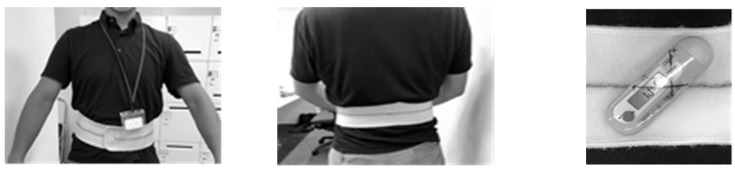
The belt was worn with reference to the navel, and the accelerometer was attached to the lumbar spine.

We measured acceleration in STS movement exercises with reference to previous studies [26,28,29,30]. After explaining the sitting posture and movement pattern of the STS movements, participants sat on a chair at a standard height (40 cm) and were placed in a sitting posture using a triaxial accelerometer (TANITA Co., Ltd. Tokyo, Japan) attached to their waist. In this study, the belt was wrapped around the navel as a reference, and the accelerometer was attached to the lumbar spine (Figure 1). Anatomy textbooks indicate that the navel is located at the height between the third and fourth lumbar vertebrae [31], and a review of chair stand evaluation methods using sensors reported that the most common sensor location was the lumbar spine (57%) (L3~L5), so the accelerometers were also attached to the lumbar spine in that study [8]. Therefore, accelerometers were installed at the lumbar spine in this study as well. After the accelerometers were installed, we confirmed that the accelerometers did not move and conducted a rise test. In the sitting posture, the participants sat with their legs hip-width apart, arms crossed in front of their chest, back straightened perpendicular to the floor, and the ankle joints held at 90°. In the sitting posture, the participant stood up quickly with maximum effort after receiving a signal from the test person, held the upright posture for approximately 2 s, sat down at normal speed, and held the sitting posture for approximately 2 s. The sampling period of the triaxial accelerometer (±8 G) was 128 Hz.

##### Data Acquisition

The data obtained from the sensors were transferred to a laptop and filtered by applying a low-pass Butterworth filter (cutoff frequency = 6 Hz) in MATLAB (The Mathworks, Inc., Natick, MA, USA; version 9.22).

##### Acceleration Parameters

**Figure 2:**
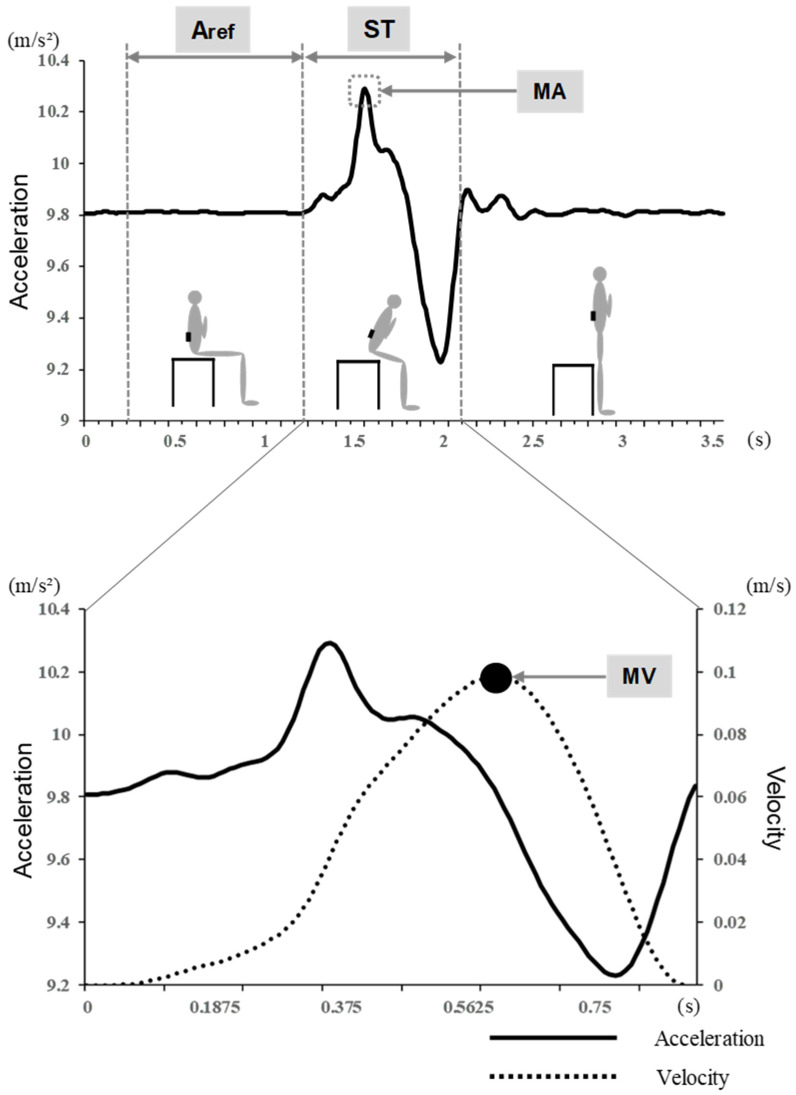
Acceleration parameters in STS movement. Aref: Acceleration Reference. ST: Stand-up Time. MA: Maximum Acceleration. MV: Maximum Velocity.

The acceleration parameter was converted to composite acceleration based on previous studies [28,29,30] and corrected as follows, with four parameters calculated (Figure 2).

$$\begin{array}{c} Acorr\;(relative\;value\;of\;acceleration)\\ \;\;\;\;\;\;\;\;\;\;\;\;\;\;\;\;\;\;\;\;\;\;\;\;\;=Ameans\;(measured\;value\;of\;acceleration)\;–\;Aref\;(reference\;value)+9.81 \end{array}$$

1. Maximal Acceleration (MA): maximum acceleration in STS movement.
2. Maximal Velocity (MV): The maximum velocity in the STS maneuver was calculated by integrating the acceleration, assuming that the velocity at the start of STS was 0 m/s.
3. Maximal Power (MP): Maximum power during rising motion. First, muscle power (F) was calculated by fitting it to the following formula: F=m×Acorr, where m indicates the body weight. Next, muscle power (P) was calculated by multiplying the muscle force (F) by velocity (v): P=F/v, which corresponds to the maximum value obtained by multiplying the solid and dotted lines in Figure 2 (bottom) by body weight.
4. Stand -up time (ST): Based on previous studies, the start of the stand-up motion was calculated using the differential acceleration value (Figure 2). The end of the standing motion was defined as the first sample in which acceleration reached the reference value after the minimum value was recorded.

#### 2.2.2. Performance Test

##### Five-Time Sit-to-Stand Test

The five-time STS test was measured according to a previous study [32]. The participants were asked to rise from a chair of standard height (40 cm) five times as fast as possible with their arms folded. The shorter time of the two trials was used for analyses. A review of previous studies has also shown its validity and reliability as an indicator for assessing lower extremity muscle strength in the older population [33].

##### Timed up and Go

The timed up and go (TUG) test is a commonly used performance test to evaluate functional mobility or dynamic balance [34]. The distance between the chair and marker was 3 m. According to the modified method, participants were instructed to rise from the chair, turn around a marker, and sit down on the chair as fast as possible [32].

##### One-Leg Balance with Eyes Open

In measuring one-leg balance with eyes open, we recorded the length of time during which participants were able to stand on one leg, up to a maximum of 60 s, to evaluate static balance. Participants were allowed to choose the lifted leg. The longest time of two trials was used for analysis [32]. Good reliability has been confirmed in older adults [35].

##### Five-Meter Habitual Walk

In measuring 5-meter habitual walk, we created an 11-meter walking path with a 5-meter central part for measurements. Participants were asked to walk at their usual pace along the walking path two times, with the shorter time being recorded. Systematic reviews have reported good reliability with this test and that it is a useful predictor of functional decline and death [36].

##### Grip Strength

To measure grip strength, participants were asked to demonstrate grip strength by squeezing a hand dynamometer (TKK5401, Takei Scientific Instruments Co. Ltd., Niigata, Japan) with one hand after the other. The highest score of two trials for each hand was used for analysis [32]. Prior research has shown that grip strength is useful in assessing muscle strength in older adults [37].

#### 2.2.3. Mobility Limitation

Mobility limitation was investigated according to Visser et al. [38] and Tsuji et al. [21]. Three activities were investigated: ascending and descending stairs, rising from a chair, and walking for 15 min. The survey consisted of three questions: “Do you climb stairs without holding onto a handrail or wall?”, “Do you stand up from a chair without holding onto anything?”, and “Do you walk continuously for about 15 min? All required a “yes” or “no” response, and those who answered “yes” were categorized as “good” and those who answered “no” as “poor”, respectively. Those who were “poor” in even one of the three movements were categorized as “mobility limitation”.

### 2.3. Sample Size

The epidemiological survey in this study was a multipurpose cohort and was not designed specifically for this study; therefore, no sample size was estimated.

### 2.4. Statistical Analyses

For comparisons of subjects by sex and by the participants with and without assessment of reliability, continuous variables were compared using the unpaired t-test, and categorical variables were compared using the χ^2^ test.

The intraclass correlation coefficient (ICC) was calculated to examine test-retest reliability. As the sample was measured twice by a single person, the ICC (two-way mixed model, type consistency) was calculated for the one-way randomization model. Based on the test between trials by analysis of variance, if no significant trial differences were found, the obtained ICC was considered significant; an ICC of 0.70 or higher was considered good [39].

Pearson’s correlation analysis was performed separately for men and women to examine the relationship between the acceleration parameters and performance tests. Un-paired t-test and an analysis of covariance were used to compare the presence or absence of mobility limitation. Adjustments were made for sex, age, BMI, presence or absence of low back pain, and presence or absence of knee pain. For each comparison, Cohen’s d [30,31,32,33,34,35,36,37,38,39,40] was calculated as the effect size, which indicates the magnitude of the difference. In this study, 0.2 was considered small, 0.5 moderate, and 0.8 large, and these values were used based on previous studies [40]. SPSS (version 26.0; IBM Corp., Armonk, NY, USA) was used for statistical analyses. The statistical significance level was set at *p* < 5% for all cases.

## 3. Results

### 3.1. Descriptive Data of Participants

Table 1 shows the basic demographics, acceleration parameters, performance tests, and exercise limitations of the participants by sex. Among the 244 participants, 43.9% were male and 56.1% were female. The mean ± SD age was 77.4 ± 4.7 years for males and 75.6 ± 5.3 years for females, with males being significantly higher. For the acceleration parameters, significant sex differences were found for MV and MP, with men having greater values (shorter ST) than women for all four parameters. In performance tests, significant differences were found only in grip strength. A higher percentage of women (43.8%) than men (30.8%) had mobility limitations, although no sex differences were observed.

### 3.2. Test-Retest Reliability of Acceleration Parameters

Table 2 lists the physical characteristics of the 12 participants in the reliability study and the values measured during the first acceleration measurement trial. No significant differences were found between the 232 who did not participate in the reliability study and the 12 who did.

Table 3 shows the measured values of each parameter in the two trials of acceleration measurement and the ICC based on them, as well as the results of the comparison of the differences between the two trials. ICCs of 0.70 or higher were obtained, which is considered good for all parameters (MA = 0.761, MV = 0.772, MP = 0.894, ST = 0.714), confirming excellent reliability.

### 3.3. Relationships among Acceleration Parameters and Performance Tests

Table 4 lists the Pearson’s correlation coefficients between each acceleration parameter and the performance tests. Significant associations were found between many items in both men and women. For the acceleration parameters, MA, MV, and MP were relatively strongly associated with the 5-time STS test (men: r = −0.36~−0.47; women: r = −0.37~−0.45) and the timed up and go test (men: r = −0.39~0.47, women: r = −0.43~−0.51). MP was also strongly associated with grip strength (men: r = 0.48, women: r = 0.43). The ST showed a relatively weak association (r < 0.3) with a small correlation coefficient compared to the other acceleration parameters.

### 3.4. Relationships among Acceleration Parameters and Mobility Limitations

Table 5 presents the results for each acceleration parameter, based on the presence or absence of orthostatic mobility limitations. Comparisons based on the presence or absence of mobility limitations confirmed significant associations for all of the parameters in the unadjusted model. After adjustment, all parameters showed significance, except for ST. Regarding effect sizes, which indicate the magnitude of differences, small effect sizes (MA = 0.41, MV = 0.48, MP = 0.46) were identified for all parameters, with a relatively low value (d = 0.30) for ST compared to the other parameters.

## 4. Discussion

This study was designed to be more representative of the older population by using an epidemiological survey of more than 100 randomly sampled individuals from the community rather than a laboratory setting, and to examine the relationship between acceleration in STS movement and physical function by gender. In addition, we examined the test-retest reliability of the acceleration in STS movements. When the reliability of acceleration in STS movement was examined, good reliability was observed for all acceleration parameters. In relation to physical function, most items showed significant associations, with relatively strong associations between 5-time STS test and TUG for both men and women. In addition, women tended to show stronger associations with the items than men. In relation to mobility limitations, significant associations were shown for all acceleration variables except ST, even after adjustment for confounders. We believe that the results obtained in this study will be a valuable resource for the development of field tests for acceleration in STS movement.

The acceleration parameters used in this study showed excellent reliability, with ICCs of 0.70 or higher, which were judged to be good [39] for all of the parameters (Table 3). The reliability of acceleration parameters in STS movements in older participants has been examined in previous studies [25,41], and excellent reliability has been confirmed. Although there were differences between the previous study and this study in the measurement equipment and the method used to calculate acceleration parameters, and the sample size was small (12 subjects), the fact that good ICCs were found confirms the reliability of the evaluation of acceleration in STS movement itself.

Low to moderately significant correlation coefficients were found between acceleration (MA, MV, MP) in STS exercise and several field tests for both men and women. Relatively strong associations were also found with TUG, except for the 5-time STS test, which were measured using chair rise movements as well as acceleration measurements. The results support previous studies that have reported significant associations between acceleration in STS movement and the 5-time STS test and TUG [13]. On the other hand, the correlation coefficients obtained in this study were slightly lower than those in the previous studies [13]. Although a moderate association with TUG was confirmed in this study as well, the correlation coefficient was slightly lower when compared to previous studies. This may be explained by the characteristics of the subjects. The previous study included 36 older subjects aged 70 years and older, and the average age of the subjects was 82.2 years. In contrast, the present study was conducted on adults aged 65 years and older, with an average age of 77.4 years for men and 75.6 years for women. Glenn et al. divided adults aged 18~97 into four groups and investigated power during chair rise, and found that the trend of decline was weak during the 70s, but declined sharply after the age of 80 [42]. In other words, it is thought that the older the age, the more acutely the lower limb muscle strength can be evaluated, and the acceleration in STS movement in this study was also affected by this, which is why it showed a lower correlation coefficient than in previous studies [13].

In MP, both men and women showed moderate correlation coefficients with grip strength. Despite differences in the methods and equipment used to calculate muscle power, previous studies [43,44] have reported a significant association between power and grip strength in STS movements, and the results of the present study support these findings. Since lower limb muscle power in older adults has been associated with changes in grip strength [45], and the STS movement test has been considered a performance test to assess overall physical function in older adults, along with grip strength and gait tests [46], we believe that a relatively strong association between MP and grip strength was confirmed in this study. In addition, MP showed significant associations with many items in this study, including the 5-time STS test (lower limb muscle strength), TUG (dynamic balance), and 5 m walking time (mobility), which may reflect the overall physical function of older adults.

In addition, more items were significantly associated with acceleration parameters and field tests in women than in men, and more items tended to show relatively strong associations (Table 4). This trend supports the report that the associations between lower limb muscle strength, lower limb muscle power, and physical function tests, such as gait speed and TUG, are stronger in women than in men [21,47]. On the other hand, to the authors’ knowledge, no studies have examined the relationship between acceleration in STS movements and physical function by gender. This is because the nature of the studies dealing with physical fitness data on acceleration is thought to be mainly laboratory-based validation, and there is a lack of studies validating the usefulness of physical fitness measurement and evaluation methods based on community-based epidemiological surveys, such as in the present study. Therefore, the findings from the epidemiological survey in this study provide important material for developing acceleration in STS movements into a field test.

As shown in Table 5, small effect sizes were identified for all parameters between the presence and absence of orthostatic mobility limitation, and significant associations were still found for MA, MV, and MP even after adjusting for sex, age, and BMI. The association between mobility, lower limb muscle strength, and power, assessed using a questionnaire-based survey, has been previously reported [21,48,49]. MA and MV, which directly evaluate the speed of the rising motion, and MP, which estimates muscle power based on these parameters, were more strongly related to lower -limb muscle strength and lower -limb muscle power than ST, a time parameter, in the acceleration parameter in the STS movement evaluated in this study [15,26]. In addition, it has been reported that lower limb muscle power is closely related to smooth performance of activities of daily living in older adults, and the results of the association between MA, MV, and MP and the presence or absence of mobility limitations are reasonable. In addition to the physical functions objectively evaluated by the performance test, the acceleration parameters (MA, MV, and MP) were found to be related to the ability to move based on the subjective assessment of older participants.

This may provide valuable data to support the usefulness of acceleration assessment as a field test to evaluate acceleration in STS movements. However, this study has some limitations. The first limitation of this study is the possibility of selection bias. In this study, we succeeded in increasing the representativeness and sample size of the subjects by conducting random sampling from the Basic Resident Registers. On the other hand, the findings of this study may not necessarily be applicable to more frail older adults, such as those certified as requiring support or nursing care. Therefore, we believe it is necessary to conduct future studies with a wider range of physical frailty levels. Second, as this was a cross-sectional study, causal relationships could not be established. As this study was based on baseline measurements from a prospective study, future studies should follow these participants longitudinally to determine whether acceleration parameters are useful in predicting the occurrence of mobility limitations. Third, the acceleration parameters were limited. Although the acceleration parameters in this study were calculated with reference to previous studies, they were by no means numerous, as only three-axis accelerometers were used in this study. Future studies using an angular velocity system or other devices to examine acceleration by axis (mediolateral and anterior–posterior) may provide more information and useful findings on acceleration in STS movements in the older adult population.

## 5. Conclusions

The acceleration parameters in STS movement showed good reliability and validity as measures for assessing lower limb function in older adults. MA, MV, and MP were strongly associated with the 5-time STS test and TUG test results and the ability to perform standing and other physical movements. MP was also strongly related to grip strength, indicating that it may be useful as a measure of muscle strength assessment in older adults. These findings support the usefulness of sensor-based measurements of STS movement, which are expected to be useful in various care settings.

## Figures and Tables

**Table 1:** Characteristics of participants.

	Men	Women	
N (%)	107 (43.9)	137 (56.1)	
<Characteristics>			
Age (years), mean ± SD	77.4 ± 4.7	75.6 ± 5.3	*
Height (cm), mean ± SD	163.6 ± 5.6	151.6 ± 5.1	*
Body weight (kg), mean ± SD	63.2 ± 8.9	51.2 ± 7.5	*
BMI (kg/m^2^)			
<18.5	5.6% (6)	9.5% (13)	
18.5~24.9	65.4% (70)	72.3% (99)	
≥25	29.0% (31)	18.2% (25)	
Lower back pain ^a^, yes % (n)	33.6% (36)	28.5% (39)	
Lower limb pain ^a^, yes % (n)	6.5% (7)	20.4% (28)	
<Acceleration parameters>			
MA (m/s^2^), mean ± SD	10.25 ± 0.17	10.21 ± 0.15	
MV (m/s), mean ± SD	0.11 ± 0.02	0.09 ± 0.02	*
MP (W), mean ± SD	69.62 ± 17.39	45.85 ± 13.91	*
ST (s), mean ± SD	1.09 ± 0.16	1.10 ± 0.18	
<Physical performance test>			
5-time STS (s), mean ± SD	7.00 ± 2.19	6.61 ± 1.81	
Timed up and go (s), mean ± SD	5.71 ± 1.26	5.66 ± 1.09	
One-leg balance with eyes open (s), mean ± SD	31.82 ± 22.28	36.01 ± 21.74	
5-meter habitual walk (s), mean ± SD	3.59 ± 0.66	3.46 ± 0.52	
Grip strength (s), mean ± SD	34.13 ± 6.04	22.79 ± 3.93	*
<Self-reported mobility limitations>			
Climbing 10 steps ^a^, difficult % (n)	22.4% (24)	32.8% (45)	
Rising from chair ^a^, difficult % (n)	8.4% (9)	14.6% (20)	
Walking for 15 minutes ^a^, difficult % (n)	7.5% (8)	13.1% (18)	
Mobility limitations ^a^, incident % (n)	30.8% (33)	43.8% (60)	

* *p* < 0.05 (presence of gender difference). ^a^: χ^2^ test. SD: standard deviation. BMI: body mass index. MA: maximum acceleration, MV: maximum velocity, MP: maximum power, ST: stand up time.

**Table 2:** Comparison of characteristics and acceleration parameters between participants with and without assessment of reliability.

	Participants with Assessment of Reliability (n = 12)	Participants without Assessment of Reliability (n = 232)	*p*-Value
<Characteristics>			
Age (years), mean ± SD	77.0 ± 4.0	76.4 ± 5.2	0.676
Percentage of women ^a^ % (n)	50.0 (6)	56.5 (131)	0.768
Height (cm), mean ± SD	156.9 ± 7.1	156.8 ± 8.0	0.992
Body weight (kg), mean ± SD	54.6 ± 7.6	56.6 ± 10.2	0.512
BMI (kg/m^2^) ^a^ % (n)			
<18.5	8.3% (1)	7.8% (18)	
18.5~24.9	83.3% (10)	68.5% (159)	0.463
≥25	8.3% (1)	23.7% (55)	
Lower back pain ^a^, yes % (n)	50.0% (6)	29.7% (69)	0.196
Lower limb pain ^a^, yes % (n)	8.3% (1)	14.7% (34)	0.465
<Acceleration parameters>			
MA (m/s^2^), mean ± SD	10.26 ± 0.13	10.23 ± 0.16	0.477
MV (m/s), mean ± SD	0.11 ± 0.02	0.11 ± 0.03	0.160
MP (W), mean ± SD	59.23 ± 13.87	56.12 ± 19.75	0.590
ST (s), mean ± SD	1.05 ± 0.10	1.10 ± 0.18	0.934

^a^: χ^2^ test, SD: standard deviation, ns: not significant. BMI: body mass index. MA: maximum acceleration, MV: maximum velocity, MP: maximum power. ST: stand up time.

**Table 3:** Test-retest reliability of acceleration parameters.

		Test1	Test2	ICC	F	*p*-Value
		Mean SD	Mean SD			
MA	(m/s^2^)	10.26 ± 0.13	10.23 ± 0.12	0.761	1.523	0.243
MV	(m/s)	0.11 ± 0.02	0.11 ± 0.02	0.772	0.353	0.565
MP	(W)	59.23 ± 13.87	58.06 ± 14.61	0.894	0.363	0.559
ST	(s)	1.05 ± 0.10	1.06 ± 0.12	0.714	0.132	0.723

n = 12. SD: standard deviation, ICC: intraclass correlation coefficient, MA: maximum acceleration, MV: maximum velocity, MP: maximum power, ST: stand up time.

**Table 4:** Pearson correlation coefficients between acceleration parameters and physical performance tests.

		Acceleration Parameters			
		MA	MV	MP	ST
**<Men>**					
5-time STS test	(s)	−0.36 *	−0.43 *	−0.47 *	0.24 *
Timed up and go	(s)	−0.39 *	−0.44 *	−0.47 *	0.27 *
One-leg balance with eyes open	(s)	0.29 *	0.19	0.15	−0.13
5-meter habitual walk	(s)	−0.21 *	−0.34 *	−0.39 *	0.17
Grip strength	(kg)	0.22 *	0.35 *	0.48 *	−0.24 *
**<Women>**					
5-time STS test	(s)	−0.45 *	−0.44 *	−0.37 *	0.17 *
Timed up and go	(s)	−0.43 *	−0.51 *	−0.43 *	0.10
One-leg balance with eyes open	(s)	0.33 *	0.25 *	0.16	−0.21 *
5-meter habitual walk	(s)	−0.32 *	−0.36 *	−0.32 *	0.11
Grip strength	(kg)	0.34 *	0.29 *	0.43 *	−0.15

* *p* < 0.05 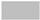 |Pearson-*r*| ≥ 0.4 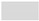 0.4 > |Pearson-*r*| ≥ 0.3 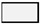 0.3 > |Pearson-*r*|. MA: maximum acceleration, MV: maximum velocity, MP: maximum power, ST: stand up time.

**Table 5:** Comparison of acceleration parameters between with and without mobility limitation.

		No (n = 151)	Yes (n = 93)	*p*-Value	Effect Size (Cohen’s *d*)	Adjusted *p*-Value ^a^	Adjusted *p*-Value ^b^
		Mean SD	Mean SD				
MA	(m/s^2^)	10.25 ± 0.16	10.19 ± 0.15	0.002	0.41	0.015	0.012
MV	(m/s)	0.10 ± 0.02	0.09 ± 0.03	<0.001	0.48	0.011	0.010
MP	(W)	59.63 ± 19.19	50.82 ± 18.82	<0.001	0.46	0.032	0.014
ST	(s)	1.07 ± 0.18	1.12 ± 0.16	0.027	0.30	0.063	0.069

*p*-value: unpaired *t* test. ^a^: ANCOVA models adjusted for age and gender. ^b^: ANCOVA models adjusted for BMI, lower back pain and lower limb pain, in addition to “a”. SD: standard deviation, ns: not significant, MA: maximum acceleration, MV: maximum velocity, MP: maximum power, ST: stand up time.

## Data Availability

The data that support the findings of this study are available on request from the corresponding author. The data are not publicly available due to privacy or ethical restrictions.
